# Bioaerosols and Airborne Transmission in the Dental Clinic

**DOI:** 10.1016/j.identj.2024.09.026

**Published:** 2024-11-06

**Authors:** James R. Allison, Stefanie Tiede, Richard Holliday, Justin Durham, Nicholas S. Jakubovics

**Affiliations:** a Faculty of Medical Sciences, School of Dental Sciences, Newcastle University, Newcastle upon Tyne, UK; b Newcastle upon Tyne Hospitals NHS Foundation Trust, Newcastle upon Tyne, UK; c Bundeszahnärztekammer e.V., Berlin, Germany

**Keywords:** Aerosol, Bioaerosol, Aerosol generating procedure, Airborne transmission, Ventilation, Infection control, Dental, Dental equipment, Fallow time

## Abstract

The importance of aerosols (particles suspended in air) produced during dental procedures became more apparent than ever during the COVID-19 pandemic. Concerns over transmission of infection in these aerosols led to unprecedented disruption to dental services across the world, adversely impacting patients’ oral health. This article discusses the evidence related to airborne transmission of infectious diseases and the relevance to dentistry. The production of bioaerosols (aerosols carrying biological material) during dental procedures is explored, as well as how the potential risks posed by these bioaerosols can be controlled. A better understanding of dental bioaerosols is needed to prevent similar disruption to dental services in future outbreaks, and to reduce the risk of infection of dental professionals when treating patients with active infections who require urgent or emergency dental care.

## Introduction

**Fig. 1:**
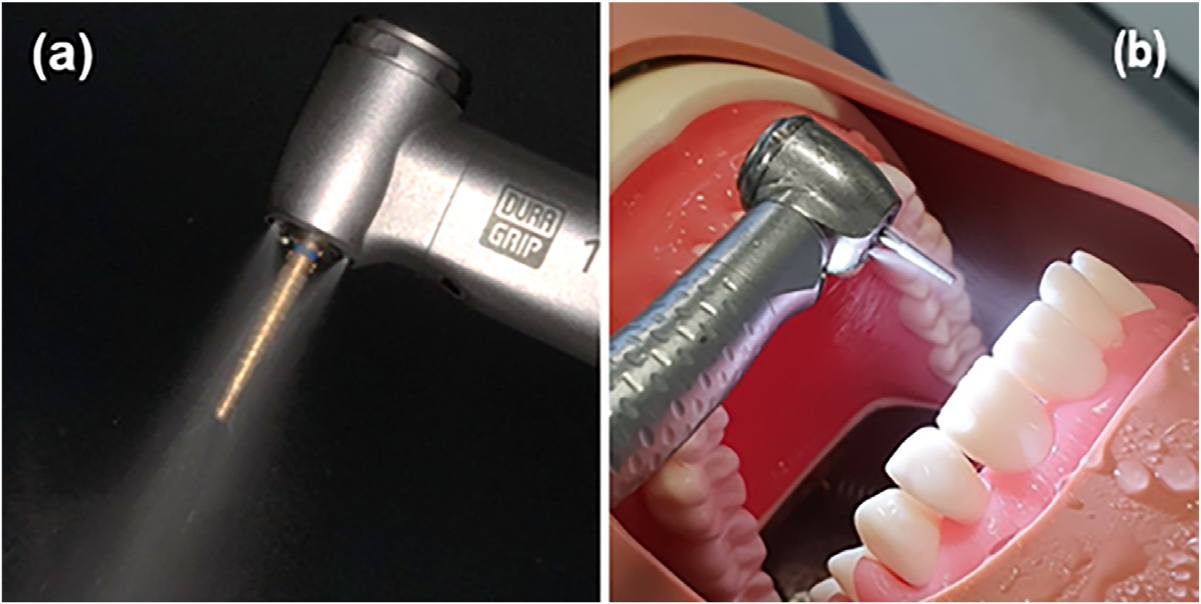
Aerosols produced by an air-turbine handpiece (A) adapted from Allison et al,^95^ and (B) during a simulated procedure in a dental mannequin.

In early 2020, the world experienced unprecedented disruption due to the evolving coronavirus disease 2019 (COVID-19) pandemic. Usual dental services in many countries were either suspended or severely limited due to concerns over transmission of the disease in dental clinics.^1^ The main concern in dentistry during this time was the risk of dispersion of the causative virus, severe acute respiratory syndrome coronavirus 2 (SARS-CoV-2), from patients’ saliva in aerosols – tiny liquid droplets which can remain suspended in the air – produced by dental instruments such as dental drills and scalers during dental treatment (Figure 1). The challenge was not so much that there was a known risk, but rather that the level of risk was unknown. The result was that millions of patients missed out on dental treatment, worsening their oral health outcomes.^2^^,^^3^ This concern was also present elsewhere in healthcare, with aerosols generated during anaesthesia, endoscopy, and surgery posing a perceived risk to patients and health professionals.^4^ Procedures which are thought to carry a risk of aerosolising infectious fluids such as blood, saliva, or respiratory secretions in *bioaerosols* (aerosols containing biological material), are termed ‘aerosol generating procedures’ (AGPs).

The AGP became an important concept during the SARS outbreak in 2002-2004,^5^^,^^6^ and later during the influenza A H1N1 pandemic in 2009.^7^ The World Health Organisation (WHO) defines high-risk AGPs as ‘medical procedures that have been reported to be aerosol-generating and consistently associated with an increased risk of pathogen transmission’.^8^ Recent reviews of the evidence surrounding bioaerosols and AGPs in healthcare conclude that the existing evidence is heterogenous and of mixed quality,^4^^,^^9^, ^10^, ^11^ however, dental procedures using high-speed instruments are classed as high-risk AGPs.^11^^,^^12^ Thus, dental health professionals need to exercise special care when treating patients with airborne infectious diseases and need to be well educated in the prevention and control of transmission of these diseases.

### Respiratory aerosols and airborne transmission of disease

Aside from the aerosols created during specific medical or dental AGPs, normal respiratory activities such as breathing, talking, and coughing also generate aerosols, and the amount of aerosol produced is related to the degree of respiratory effort. For example, talking produces 35 times more respiratory aerosol, and coughing 370 times more, than quiet breathing.^13^ These aerosols are generated within the respiratory tract by the interaction of turbulent airflows with the films of liquid and mucus which line the airways.^14^^,^^15^ The presence of microorganisms in these fluids means that microbes can be carried within respiratory aerosols.

There is now overwhelming evidence that airborne transmission of several diseases occurs by inhalation of microbe-laden aerosols.^16^ For example, infectious virus, such as SARS-CoV-2,^17^^,^^18^ Middle East respiratory virus (MERS),^19^ influenza,^20^^,^^21^ and respiratory syncytial virus (RSV),^22^ as well as nucleic acids from SARS-CoV-1,^23^ rhinovirus,^24^ and measles virus^25^ can be recovered in air samples from infected patients. Furthermore, experimental^26^ and epidemiological data from humans^27^, ^28^, ^29^, ^30^, ^31^, ^32^, ^33^ and data from animal studies^34^, ^35^, ^36^, ^37^, ^38^ confirm a substantial role for these infectious aerosols in transmitting clinical disease. Bacterial diseases, such as pulmonary tuberculosis, caused by *Mycobacterium tuberculosis*, can also be spread by airborne transmission, as demonstrated in experiments where guinea pigs exposed to air from hospital tuberculosis wards, but not unexposed animals, became infected.^39^^,^^40^ Additionally, viable bacterial pathogens such as *Pseudomonas aeruginosa, Staphylococcus aureus, Stenotrophomonas maltophilia, Achromobacter* spp., and *Burkholderia* spp. have also been isolated from air samples during coughing in patients with cystic fibrosis at distances up to 4 m, and up to 45 minutes after coughing.^41^ Airborne transmission of the gram-negative coccobacillus *Bordetella pertussis*, which causes whooping cough, has also been demonstrated in a non-human primate model between inoculated animals and animals housed in separate cages at a distance of 2 m.^42^ In rare cases, epidemiological evidence also exists for airborne transmission of pneumonic plague, caused by the gram-negative coccobacillus, *Yersinia pestis*.^43^ Additionally, the presence of DNA from the fungal pathogen *Pneumocystis jirovecii* has also been demonstrated in the air surrounding patients with *Pneumocystis* pneumonia, and at distances of up to 8 m.^44^^,^^45^ Aside from human-to-human transmission via respiratory aerosols, there have been isolated reports of airborne transmission of zoonotic bacterial pathogens from animals to humans, such as *Francisella tularensis*^46^^,^^47^ and *Burkholderia pseudomallei*.^48^

## The fate of aerosols

Once in the environment, aerosols do not remain stable. The momentum with which they are emitted, either during breathing, coughing, or via an instrument such as a dental drill, affects how long they remain in the air and how far they travel.^49^ The size of aerosol particles also dictates their residence time, with larger particles settling to the ground more quickly than smaller ones. This relationship is described by Stokes’ law, and whilst in still air, a spherical 100 μm-diameter particle might take 5 seconds to settle to the ground from a height of 1.5 m, for a 5 μm particle, this would take 33 minutes.^16^ The temperature and relative humidity of the environment also influences the aerosol, as both affect the rate of evaporation. Smaller particles evaporate more quickly, and therefore become smaller and remain suspended for longer. The ultimate size and composition of the resulting particle is also determined partly by their salt, protein, surfactant, and water content.^50^^,^^51^

One persisting problem in the literature relating to airborne transmission and AGPs is the conventional infection prevention and control (IPC) dogma that infectious diseases are transmitted via 1 of 3 clearly defined, and mutually exclusive routes: the contact route, whereby infection is transmitted by direct contact with the patient or their surroundings; the droplet route, where transmission occurs over short distances via the patient's respiratory droplets coming into contact with an individual's mucosa; or the airborne route, which can occur over larger distances, via aerosols (typically defined as particles <5 µm diameter) from the patient's respiratory tract being inhaled or contacting an individual's mucosa.^12^ More recently, however, several authors have questioned the validity of these neat delineations and have highlighted that the literature on which they are based has been misinterpreted.^52^ In fact, particles much larger than 5 µm – up to 100 µm – can remain suspended in the air and travel further than 2 m.^53^

**Fig. 2:**
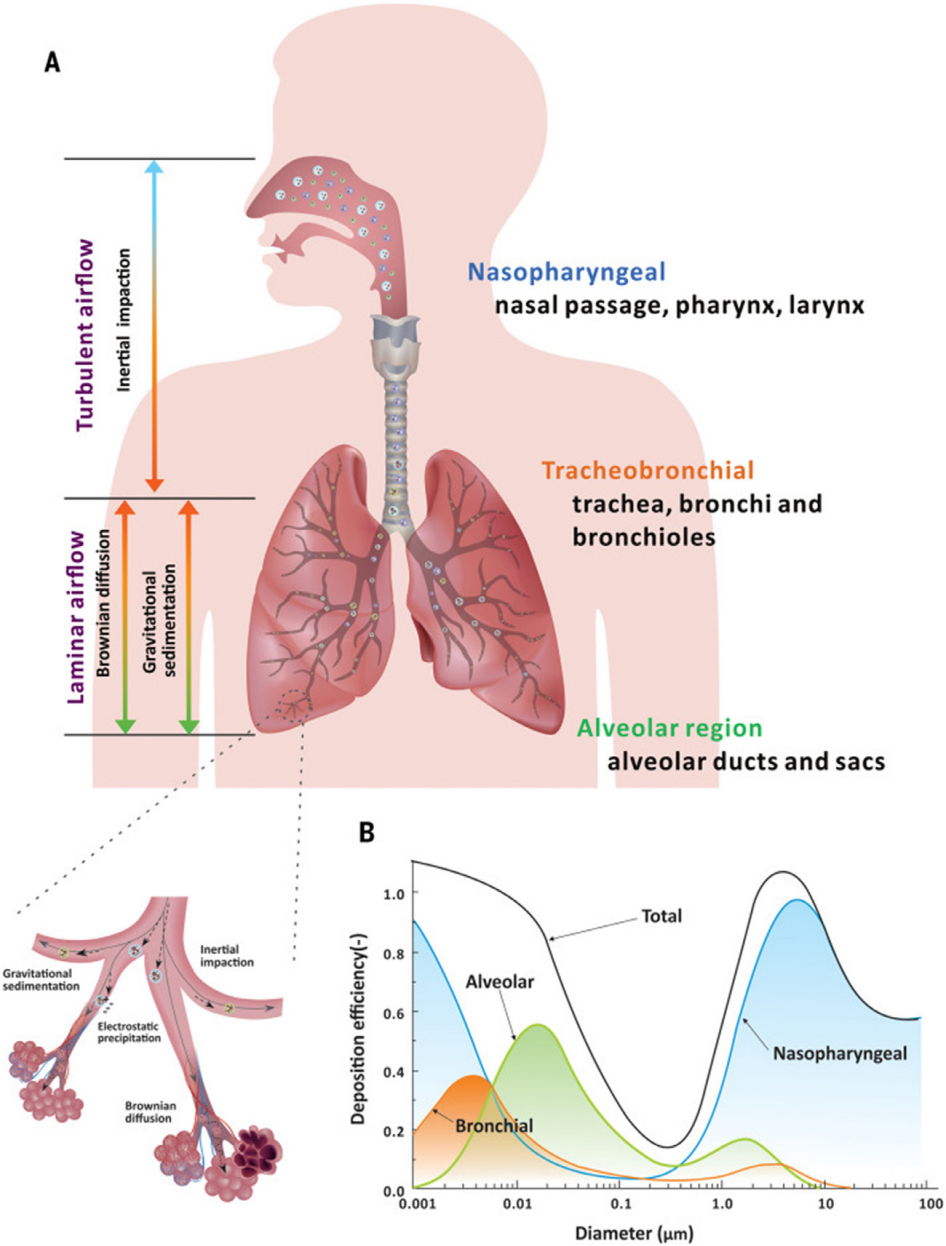
Mechanisms of aerosol deposition in the respiratory tract, reproduced from Wang et al.^16^ (A) Mechanisms of deposition in different areas of the respiratory tract, including inertial impaction (larger aerosols), gravitational sedimentation and Brownian diffusion (smaller aerosols). (B) Deposition efficiency in different regions of the respiratory tract as a function of aerosol diameter, based on the International Committee of Radiological Protection (ICRP) lung deposition model.^146^

Once aerosolised, microbe-laden aerosols up to 100 µm can be inhaled, and these larger droplets can be deposited on exposed (eg, oral, pharyngeal, ocular) mucous membranes, potentially initiating an infection. The size of these particles dictates where in the respiratory tract they are most likely to be deposited (Figure 2). Larger aerosols of >5 µm primarily deposit in the nasopharyngeal airways, and although <5 µm aerosols may also become deposited in the upper airways, these aerosols can penetrate more deeply into the bronchial and alveolar airways. Very small aerosols <0.1 µm are primarily deposited in bronchiolar and alveolar regions.^54^ The site of deposition is important, as evidence from animal models suggests that more severe disease is caused by deposition in the smaller airways.^55^

### Bioaerosols in dentistry

As in any setting where health professionals come into close contact with patients, dental professionals are exposed to patients’ respiratory aerosols, and any microbes contained therein. In dentistry, however, aerosols are also generated by instruments such as air-turbine handpieces and ultrasonic scalers used within the microbially-laden oral cavity. Some of the first detailed studies of bioaerosols in dentistry were conducted in the 1960s and early 1970s, following the introduction of the air-turbine handpiece in the late 1950s, due to concerns surrounding aerosolised bacteria, tooth debris, and lubricating oil from these handpieces.^56^, ^57^, ^58^, ^59^, ^60^ These investigators found that microbes were dispersed from patients’ mouths during dental treatment and the degree of dispersion was affected by the instrument used, varied by the location of sampling, and decayed over time.

Bioaerosols were not high on the dental infection control agenda after this initial interest, although widespread adoption of the use of gloves, facemasks, and the safe use of sharps in dentistry did gradually occur during the 1980s, in part encouraged by the HIV/AIDS pandemic and rising prevalence of hepatitis B and C.^61^ Despite these changes, dental IPC guidance often included very little information on bioaerosol risks.^62^ Some interest was seen during the SARS and MERS outbreaks in the first and second decades of the 21st century^5^^,^^63^ however it was not until the early stages of the COVID-19 pandemic that the importance of bioaerosols in dentistry became fully apparent. Many of the opportunities prior to the COVID-19 pandemic to learn lessons on the risks posed by bioaerosols were missed, and it is important that we improve our understanding of effective mitigations to prevent similar widespread disruption in future outbreaks.

The mouth contains many diverse microbial populations, mainly situated in highly organised biofilms located within several niches including mucosal, tooth, subgingival, and non-biologic surfaces, as well as in saliva. The composition of these populations varies between individuals, as well as over the life course, and can change in the presence of disease states such as dental caries and periodontitis.^64^ Bacterial species are numerous, and several hundred taxa have been identified. The most common species belong to genera including *Streptococcus, Neisseria, Haemophilus, Rothia, Veillonella*, and *Prevotella*.^65^ Aside from bacteria, archaea and fungi are also present within the oral cavity and contribute to microbial ecology, along with a wide diversity of viruses in health,^66^^,^^67^ most frequently bacteriophages (viruses which infect bacteria), but also human viruses such as herpesviruses, retroviruses, and papillomaviruses.^68^ Viruses are also present in saliva in significant quantities during active infections with viruses such as SARS-CoV-2, influenza, RSV, HIV, and hepatitis B and C.^69^, ^70^, ^71^, ^72^, ^73^ The numerous microorganisms present in the oral cavity, including viruses, may therefore be dispersed from the mouth into the environment due to interactions between dental instrument aerosols and oral fluids and biofilms. As well as the mouth, biofilms are also present within the waterlines which supply dental instruments, which contain numerous bacterial taxa from the genera *Legionella, Mycobacterium, Serratia, Pseudomonas, Coxiella, Staphylococcus* and many others.^74^, ^75^, ^76^ Dental bioaerosols are therefore highly likely to contain bacteria from these genera^75^ in addition to those which arise from the oral cavity.

There is very little high-quality epidemiological evidence on the risk of infection of dental professionals via bioaerosols in the dental clinic, however, one study which looked at SARS-CoV-2 seroprevalence in the northwest of England during the early COVID-19 pandemic, prior to widespread vaccination, found that previous COVID-19 infection was more likely in clinical members of the dental team (∼16%), compared to both non-clinical members of the team (e.g., reception staff) and the general population at that time (∼6%).^77^ This may not be surprising, given that among other professions, dentistry may have one of the highest bioaerosol exposure risks due to the combination of close proximity and frequent potential exposure to pathogens.^78^

## Measurement of dental bioaerosols

Several methods for the measurement of aerosols in the healthcare setting have been reported in the literature, for example, by detecting the microbes or microbial products arising from individual patients, quantifying the aerosols directly by physical measurement, or by detection of tracers (biological or otherwise) added to aerosols to quantify them. These methods can be used in real clinical settings or in simulation models, and each approach has its own benefits and limitations.

### Measurement of dispersed oral microbes

Some of the earliest studies of dental bioaerosols used patients’ own oral microbiota to quantify the bioaerosols produced during dental procedures. Micik^57^ used air sampling equipment to detect bacteria in aerosols during normal respiratory activities and dental procedures in a specially designed chamber. In this study, the use of an air-turbine handpiece produced 1000 times as many bacterial colony-forming units as quiet breathing, and twice as many as during a sneeze; high-volume suction reduced bacterial dispersion by 99.4%. In a similar study, bacteria dispersed from the mouth were detectable on surfaces 1.8 m away from a procedure using an air-turbine handpiece, although this was less than during a sneeze.^56^ More contemporary applications of these methods have shown that the highest concentrations of droplets and aerosols containing bacteria settle on the area of the patient's chest during dental procedures, suggesting that the zone closest to the procedure most likely carries the greatest risk of contamination.^75^ In the same study, the authors also used DNA sequencing to show that bacteria identified in dental bioaerosols came from both the oral cavity as well from dental unit waterlines.

Most studies using these methods have looked at bacteria, but a smaller number of authors have tried to detect viruses in dental bioaerosols, mainly during the COVID-19 pandemic. One study reported SARS-CoV-2 RNA on surfaces in dental surgery in 5 out of 24 cases where dental treatment was carried out on patients with COVID-19.^79^ Another study in dental clinics in Iran during a period of high community COVID-19 prevalence found SARS-CoV-2 RNA in 36% of air samples.^80^ Very little research has been done in clinical dental settings looking at viruses other than SARS-CoV-2, although one study in dental patients reported the presence of hepatitis B virus DNA in dental aerosols from 1 out of 3 hepatitis B carriers following an orthodontic debonding procedure.^81^

Whilst sampling during an infectious disease outbreak may make the recovery of any pathogen of interest more likely, most of the time patients attending the dental clinic do not have an infection, and capturing a particular pathogen becomes unlikely during periods of low community prevalence. This is the main limiting factor in studies of this kind in the dental setting, and methodological issues in the sampling and detection of microbes may further complicate this approach. Additionally, if microorganisms are sampled directly from the clinic, it may be difficult to separate the contributions of different sources, such as the oral cavity, the respiratory tract, or the water supply of the dental instruments themselves.

### Physical aerosol measurement

An alternative method is the physical measurement of aerosol particles, which assumes that where the absolute amount of aerosol is increased, the presence of potentially infectious bioaerosols is also increased. Although this may not always be a valid assumption, the main benefit of physical aerosol measurement is that the relative size of the droplets within the aerosol can be easily resolved, which as discussed, is important because size affects dispersion, persistence, and potential infectiveness.^54^

Several instruments exist for measuring aerosols, including optical particle counters which can usually detect particles 0.3-10 μm in diameter, and more sophisticated instruments which can detect particles as small as a few nanometres.^82^ Due to the convenience of physical aerosol measurement, many investigators have used these instruments to measure dental aerosols, with varying methodological rigor. One factor limiting application, however, is the presence of background aerosols in ambient air under normal conditions which affect signal-to-noise ratio and limit the ability to detect aerosols specifically from a dental procedure. Although the physical measurement of aerosols is useful and provides rich information about aerosol characteristics, the application is not without other difficulties and pitfalls, which are well-reviewed by Gregson *et al.*^83^

### Tracer studies

Many studies have measured dental bioaerosols using a tracer, i.e., a substance that can be added to a simulated system or a clinical setting to allow components of the aerosol, such as saliva or blood, to be measured. The benefit of using a tracer, particularly during a simulated procedure in a mannequin, is that high levels of repeatability can be achieved, allowing multiple parameters to be assessed in a controlled way. An obvious limitation, however, is that sources of variation such as patients’ movement, anatomy, microbiota, and their respiratory activities, often cannot be accurately modelled. Chemical tracers such as methylene blue^84^ and other dyes^85^, ^86^, ^87^ or citric acid^87^^,^^88^ have been used to detect aerosols produced during simulated dental procedures, and other authors have increased sensitivity by using fluorescent tracers like fluorescein.^89^, ^90^, ^91^, ^92^, ^93^, ^94^, ^95^, ^96^, ^97^, ^98^ In many studies, however, the tracer is added to dental instruments’ water supply, therefore showing where water from the instrument, but not necessarily saliva and potential microbes, may be transported; this is likely to overestimate microbial dispersion in aerosols. Additionally, many such studies rely on visual or photographic methods of detecting the tracer which lack sensitivity.

Non-biological tracers are helpful but do not give any biological information, such as infectivity and survival of dispersed microbes. To address this, bacterial species including *Serratia marcescens*,^59^ *Streptococcus mutans*,^99^ *Lactobacillus acidophilus*,^89^ and *Enterococcus faecalis*^100^ have been used as tracer organisms. Only a handful of studies have used viruses as tracer organisms, for example 2 studies in laboratory containment chambers demonstrated the dispersion of equine arteritis virus^101^ and human coronavirus 229E^102^ at 60 cm and 25 cm respectively. These viruses, however, for reasons of biosafety cannot easily be used in real clinical settings, and a number of authors have therefore sought to use bacteriophages (phages), which are viruses that infect bacteria and are harmless to humans, to measure viral dispersion in clinical settings. Various phages such as phi6,^103^, ^104^, ^105^ MS2,^98^^,^^106^ and phiX174^107^^,^^108^ have been used in dental simulation studies. The overall validity of the approach used may be improved by applying a combination of the above methods, as a number of authors have sought to do, particularly by adding sensitive physical aerosol measurement to a more specific approach such as recovery of microorganisms in the clinic^109^^,^^110^ or the use of a biological tracer.^98^^,^^104^

### Bioaerosol control measures

Dental bioaerosols are clearly of great importance during emerging infectious disease outbreaks, and a better understanding of the risks posed by dental bioaerosols and of effective control measures may avoid the need to close dental services in future outbreaks, as was seen during the COVID-19 pandemic. Outside of this context however, microbial dispersion is also of importance when treating patients with endemic diseases such as COVID-19, influenza, RSV, and tuberculosis. Most dental treatment in patients with an active infection can be deferred, however urgent and emergency care cannot. As discussed, little epidemiological evidence exists on the risks to dental professionals from bioaerosols, however, population measures during pandemic outbreaks such as vaccination^111^ and face mask use^112^ may be highly effective at reducing community transmission and therefore risk to dental staff. Similarly, vaccination of healthcare workers against endemic diseases like influenza may also reduce transmission risk^113^ as has been successfully implemented for blood-borne viruses such as hepatitis B.^114^

The existing evidence, as synthesised in several systematic reviews,^11^^,^^115^, ^116^, ^117^, ^118^ suggests that dental procedures which use high-speed handpieces are more likely to disperse microbes than with other instruments, and that contamination reduces with distance from, and with time after the procedure. Effective bioaerosol control measures are therefore needed to reduce exposure risk to dental professionals and other patients. A number of systematic reviews^119^, ^120^, ^121^ and guidance documents^122^, ^123^, ^124^ have examined the evidence on methods of bioaerosol control, concluding that the data are of mixed quality, with little data relating to the dispersion of viruses in dental settings. Dental bioaerosol control methods reported in the literature generally apply 1 of 3 approaches: (1) reducing the total amount of aerosol produced, (2) reducing the number of microbes within bioaerosol, and (3) capturing or removing bioaerosols.

### Reducing aerosol production

Dispersion during dental procedures requires an aerosol within which to carry microbes, and control methods which reduce the total amount of aerosol are therefore attractive. This is mainly achieved using dental instruments which produce less aerosol than the conventional air-turbine handpiece. Air-turbine handpieces drive compressed air through a turbine in the head of the handpiece, and this air is often exhausted through the front of the handpiece, towards the tooth, along with water to cool the burn, producing considerable aerosol.^125^ Alternatively, electric motors (micromotors) can be used to drive a geared handpiece, meaning water alone can be used to cool the burn, thereby producing considerably less aerosol,^95^^,^^125^^,^^126^ with fewer microbes carried within the aerosol.^103^^,^^104^

### Reducing microbial load in bioaerosols

Control measures which aim to reduce the number of microbes within bioaerosols have also been used, either by isolating the aerosol from the rest of the oral cavity, as with rubber dam, or by inactivating microbes using antiseptic mouthrinses or disinfectants in the water supplying dental instruments.

Rubber dam is a simple and inexpensive approach which has been used in dentistry for almost a century and a half.^127^ Several systematic reviews of clinical studies have concluded that rubber dam reduces dispersion of bacteria with an estimated effect of up to 99% at 1 m from the procedure, 70% at 2 m, and little effect at greater distances.^120^, ^121^, ^122^ The effectiveness of rubber dam in controlling viral bioaerosols is also demonstrated in bacteriophage tracer studies, demonstrating a 92-100% reduction in viral aerosols.^103^

Several authors have suggested the use of antimicrobial mouthrinses such as chlorhexidine, povidone iodine, and cetylpyridinium chloride in reducing microbial load in the mouth prior to dental procedures, thereby reducing the number of microbes dispersed in dental bioaerosols. Clinical studies have generally reported a 33-94% reduction in bacterial dispersion in bioaerosols when a pre-procedural mouthrinse is used.^119^^,^^121^^,^^128^ Although several studies demonstrate a substantial reduction in salivary viral loads (eg, SARS-CoV-2) following antiseptic mouthrinse use,^129^^,^^130^ the effect on viral dispersion in dental bioaerosol remains unknown. Some clinical recommendations published during the COVID-19 pandemic acknowledged this evidence base but stopped short of recommending antimicrobial mouthrinses due to the risk of adverse effects such as allergy.^122^

The addition of antimicrobial agents such as chlorhexidine, sodium hypochlorite, and hydrogen peroxide to dental instrument irrigants has been suggested, to reduce the presence of microbes in bioaerosols; these have been shown to reduce bacterial dispersion in clinical studies by 43-88%.^120^^,^^131^^,^^132^ No clinical studies in patients have examined the effect of dental unit waterline disinfectants on viral dispersion, however simulation studies in laboratory settings demonstrate the elimination of the virus in bioaerosols using sodium hypochlorite and hydrogen peroxide in irrigants.^101^^,^^102^ Bacteriophage studies in dental settings have also demonstrated a 99% reduction in infectious virus at 0.5 m with a commercially available dental waterline disinfectant.^98^

## Capturing bioaerosols

Suction devices are routinely used in dentistry to remove saliva and irrigants from patients’ mouths during treatment. Clinical studies of high-volume dental suction demonstrate up to 80-90% reductions in bacterial dispersion in bioaerosols during dental procedures, particularly at distances close to the procedure (0.3 m).^120^^,^^121^^,^^124^ This is supported by simulation studies using droplet imaging demonstrating 92% reduction,^133^ by fluorescent tracer studies demonstrating 75% reduction at 0.5 m and 67% at 1.5 m,^93^ as well as studies using a bacteriophage tracer.^105^

To capture aerosols that escape the oral cavity, local exhaust ventilation (LEV) devices may be used. LEV, often referred to in the literature as ‘extra-oral suction’ and aerosol ‘scavenging’ or ‘extraction’ systems, uses an inlet positioned close to the patient's mouth which can achieve high airflow rates (∼3,000-5,000 L/min) to remove aerosol. One clinical study found an 89-93% reduction in bacterial dispersion when LEV was placed 0.1 m from patients’ mouths compared to 0.2 m.^128^ Simulation studies using particle counting instruments^134^ and fluorescent tracers^94^ also demonstrate reductions in detectable aerosol at 0.5 m of up to 90% and 75% reduction at 2 m.

Bioaerosols can accumulate in poorly ventilated spaces, leading to an increased risk of disease transmission.^135^ There is little definitive evidence to support specific recommendations for adequate ventilation rates for dental settings, however, ventilation rates of 10 air changes per hour (ACH) have been proposed.^136^ Following a dental procedure on an infected patient, bioaerosols may persist in the air, risking infection to others. Recommendations during the COVID-19 pandemic on how long after a procedure it becomes safe to enter the room without protective equipment (so called ‘fallow time’), were based on the limited existing evidence and expert opinion. It was suggested that larger contaminated droplets are likely to settle onto surfaces within 10 minutes, with the clearance of smaller aerosols being dependent on the ventilation rate (30 minutes at 10 ACH, 60 minutes at 5 ACH for a typical dental procedure).^122^, ^123^, ^124^ These sources also suggest that fallow time may be reduced where other bioaerosol control methods are used. Where ventilation systems are poor or cannot be upgraded, there is evidence that air filtration using HEPA filters (also referred to in the literature as ‘air cleaners’, ‘purifiers’, or ‘scrubbers’) may be effective in reducing dental bioaerosol dispersion.^108^^,^^120^^,^^122^

### Personal protective equipment

The final line of defence against infectious bioaerosols is personal protective equipment (PPE). Under normal conditions, standard infection control precautions apply to dental treatment.^12^^,^^137^ In relation to PPE, this means wearing disposable gloves, eye protection, and a fluid-resistant surgical mask where exposure to blood and saliva is likely. Where patients have a known or suspected infection, however, then specific precautions (including PPE) appropriate to the route of transmission of that infection should be used; these are termed transmission-based precautions. For infections known to be transmitted via airborne transmission (eg, influenza, coronaviruses) respiratory protective equipment such as a filtering face piece 3 (FFP3) or N95 mask should be used when providing *any* care for patients, including AGPs. When performing an AGP on patients with an infection transmitted via the droplet route, respiratory protective equipment should also be worn during AGPs.^12^^,^^137^ As discussed, however, the classical contact/droplet/airborne transmission routes have been criticised for their over-simplified approach to transmission dynamics, and so a risk assessment should take place for individual patients.^16^^,^^53^^,^^138^ Outside of normal conditions, for example during an infectious disease epidemic, specific recommendations for PPE relevant to the causative agent may be given, as during the COVID-19 pandemic.^139^^,^^140^ The evidence relating to PPE is generally of low certainty, and is largely from simulation studies assessing contamination, rather than those reporting rates of infection.^141^

### The future

It is clear that airborne transmission of infection via dental aerosols is possible, and that uncertainty over appropriate ways to minimise this risk has caused significant disruption in recent years. As the incidence of emerging infectious disease outbreaks is likely to continue to increase,^142^^,^^143^ it is vital that we better understand the risks arising from dental bioaerosols, and how to control these, in order to prevent the same disruption and impact on oral health in future outbreaks, as were seen during the COVID-19 pandemic. Better research in this area is needed, as has been highlighted by other authors,^144^^,^^145^ and this should focus not only on the fundamental aerobiology but also on factors which affect the risk of transmission in the dental clinic (patient, microorganism, procedure, environmental factors), as well as the effectiveness of bioaerosol control measures.

With a greater appreciation of the potential for airborne transmission in the dental clinic, our hope is that dental professionals are better able to assess when it may be preferable to delay elective treatment in patients with active respiratory infections, and how to implement effective control measures when unavoidable urgent or emergency care is needed.

## Author contributions

James R Allison: conceptualisation, methodology, formal analysis, investigation, writing – original draft, writing – review and editing. Stefanie Tiede: conceptualisation, methodology, writing – review and editing. Richard Holliday: conceptualisation, methodology, writing – review and editing, supervision. Justin Durham: conceptualisation, methodology, writing – review and editing, supervision. Nicholas S. Jakubovics: conceptualisation, methodology, writing – review and editing, supervision.

## Conflict of interest

The authors declare that there are no conflicts of interest. JRA is supported by a Wellcome Trust Clinical Research Training Fellowship from the Wellcome 4Ward North Clinical PhD Academy and JRA, RH, JD, and NJ have received funding for research in dental bioaerosols from: the Faculties of Dental Surgery of the Royal College of Surgeons of England and the Royal College of Surgeons of Edinburgh; the British Endodontic Society; VODEX Ltd; and AerosolShield Ltd. JRA, RH, JD, and NJ are supported by the NIHR Newcastle Biomedical Research Centre (BRC), which is a partnership between Newcastle Hospitals NHS Foundation Trust, Newcastle University, and Cumbria, Northumberland and Tyne and Wear NHS Foundation Trust and is funded by the National Institute for Health and Care Research (NIHR). The views expressed are those of the author(s) and not necessarily those of the NIHR or the Department of Health and Social Care.
